# Synthesis, crystal structure and stability of a new isomer of bis­(ethyl­ene­thio­urea)di­thio­cyanatocobalt(II)

**DOI:** 10.1107/S2056989026007383

**Published:** 2026-07-23

**Authors:** Christian Näther, Aleksej Jochim

**Affiliations:** a Institut für Anorganische Chemie, Universität Kiel, Max-Eyth.-Str. 2, 24118 Kiel, Germany; University of Aberdeen, United Kingdom

**Keywords:** synthesis, crystal structure, isomerism, thermodynamic stability, cobalt thio­cyanate, ethyl­ene­thio­urea

## Abstract

The title compound, bis­(imidazolidine-2-thione-κ*S*)di­thio­cyanato­cobalt(II), [Co(NCS)_2_(C_3_H_6_N_2_S)_2_], was prepared by the reaction of cobalt thio­cyanate with ethyl­ene­thio­urea in ethanol solution. The asymmetric unit (space group *P*2_1_/*c*) consists of one cobalt cation, as well as two crystallographically independent thio­cyanate anions and two ethyl­ene­thio­urea ligands, all of them located in general positions. The metal cations are tetra­hedrally coordinated by two N-bonding anionic ligands and two ethyl­ene­thio­urea ligands into discrete complexes. These complexes are linked by N—H⋯S hydrogen bonds into layers. The IR spectrum is in agreement with the presence of a tetra­hedral coordination with N-bonding thio­cyanate anions and measurements using X-ray powder diffraction indicate that a pure crystalline phase has been obtained. The title compound represents a new isomer of Co(NCS)_2_(C_3_H_6_N_2_S)_2_, which was already reported in the literature in space group *P*1 [Mautner *et al.*, (2018 ▸). *Polyhedron***154**, 436–442]. In contrast to the title compound, in the triclinic isomer the cobalt cations are octa­hedrally coordinated and linked into chains by μ-1,3-bridging thio­cyanate anions. Solvent-mediated conversion experiments starting from a mixture of both isomers show that the title complex is the thermodynamically stable form at room temperature.

## Abstract 2

The title compound is a new isomer of Co(NCS)_2_(C_3_H_6_N_2_S)_2_. The cobalt cations are tetra­hedrally coordinated by two N-bonding thio­cyanate anions and two ethyl­ene­thio­urea ligands into discrete complexes. Solvent-mediated conversion experiments prove that the new isomer is thermodynamically stable at room temperature, whereas the known triclinic isomer with an octa­hedral cobalt coordination and a chain structure is metastable.

## 1. Chemical context

Polymorphism and isomerism are widespread phenomena in coordination chemistry (Braga & Grepioni, 2000 ▸; Barnett *et al.*, 2002 ▸; Moulton & Zaworotko, 2001 ▸). Different polymorphs or isomers are frequently found in, for example, coordination compounds based on transition-metal thio­cyanates with N-donor coligands, which might be traced back to the fact that this class of compounds shows a large structural variability, which originates in part from the different coordination modes of this anionic ligand but also from the fact that some transition-metal cations show variability in their coordination numbers (Krebs *et al.*, 2021*a* ▸; Wellm *et al.*, 2020 ▸; Böhme *et al.*, 2020 ▸; Neumann *et al.*, 2018 ▸).

For many years, we and others have been especially inter­ested in cobalt thio­cyanate and seleno­cyanate compounds with the general composition Co(NC*X*)_2_(*L*)_2_ (*X* = S, Se and *L* = N-donor coligand) because some of them show inter­esting magnetic properties such as single-chain magnet (SCM) behavior (Wöhlert *et al.*, 2012 ▸, 2013 ▸). This is the case in compounds in which the cobalt cations are octa­hedrally coordinated by two N- and two S-bonding thio­cyanate anions and two N-donor coligands and linked into chains by μ-1,3-bridging thio­cyanate anions. Such a coordination represents an *MA*_2_*B*_2_*C*_2_ system for which five isomers exist, namely one all-*trans*, three different *cis*–*cis–trans* and one all-*cis* isomer. We found that for SCM behavior to be observed in compounds with linear chains, an all-*trans* coordination or a *cis*–*cis*–*trans* coordination with the coligands in *trans*-positions is needed (Böhme *et al.*, 2020 ▸). The other isomers lead to the formation of corrugated chains, for which the magnetic exchange is suppressed (Böhme *et al.*, 2020 ▸). There are a few additional compounds with this composition in which Co(NCS)_2_ layers are observed and that show ferromagnetic ordering at low temperatures (Suckert *et al.*, 2016 ▸).

In the beginning, we focused on pyridine derivatives as coligands, for which the majority of compounds consist of linear chains. However, in the course of our systematic work we also used 4-di­methyl­amino­pyridine as coligand, but in contrast to all other ligands investigated before, discrete tetra­hedral complexes were obtained, which crystallize in different isomeric or polymorphic modifications and which represent an isomer of the compounds mentioned above (Näther *et al.*, 2018 ▸; Krebs *et al.*, 2021*b* ▸). Such complexes are of less inter­est for our project because no SCM behavior can be observed. The reason why for some ligands chains and for some others discrete complexes are observed is still unknown.

In 2018, Mautner and coworkers reported on the synthesis, structure and properties of Co(NCS)_2_(4-meth­oxy­pyridine)_2_, which shows linear chains and SCM behavior (Mautner *et al.*, 2018 ▸; Rams *et al.*, 2020 ▸). However, under slightly different reaction conditions they were also able to prepare a second isomer with this composition that consists of discrete complexes. Unfortunately it was not determined which of the two isomers is thermodynamically stable at a given temperature.

**Figure 1:**
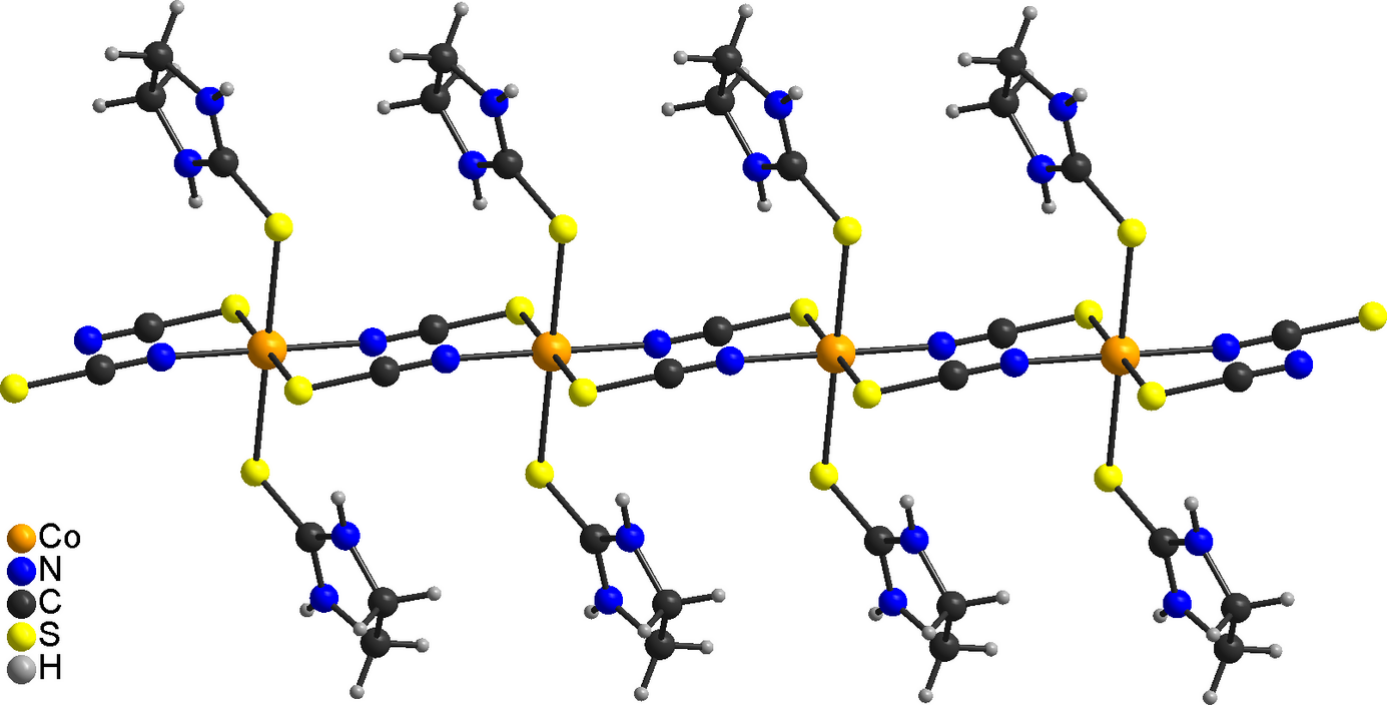
View of apart of a chain in the known isomer of Co(NCS)_2_(ethyl­ene­thio­urea)_2_.

Some time later, we became inter­ested in such compounds with coligands other than pyridine derivatives, to study the influence of the coligand on the structural and magnetic behavior. With ethyl­ene­thio­urea, we obtained a compound with the composition Co(NCS)_2_(ethyl­ene­thio­urea)_2_ (CSD refcode ZZZFAI01, space group *P*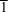; Böhme *et al.*, 2020 ▸) in which the cations are octa­hedrally coordinated with an all-*trans* coordination and linked into linear chains by the anionic ligands (Fig. 1 ▸). Recently we tried to synthesize this compound again for additional investigations, but instead of the known chain isomer, the title compound was obtained and we now describe its crystal structure and thermodynamic stability using solvent-mediated conversion experiments.
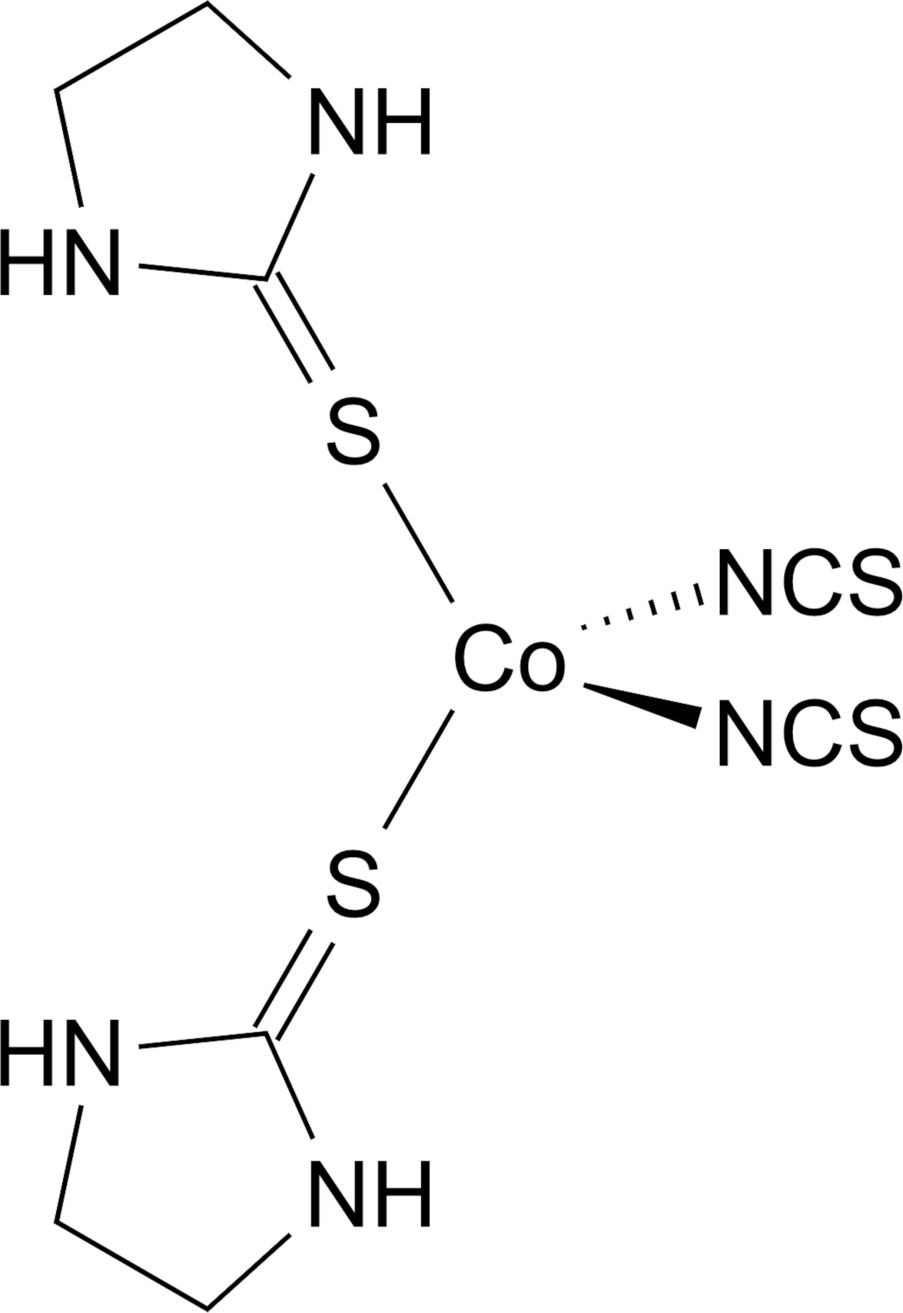


## 2. Structural commentary

**Figure 2:**
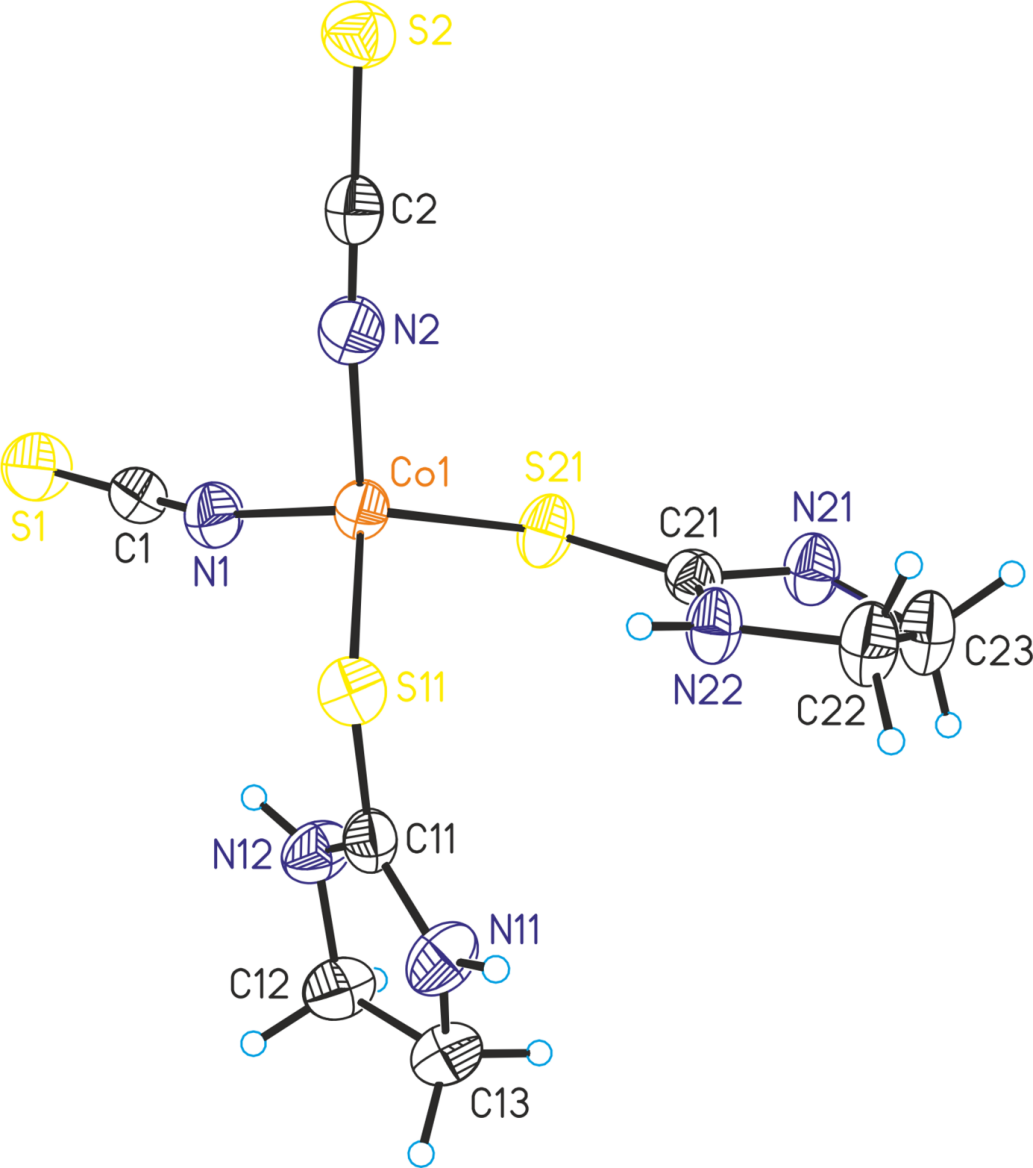
Crystal structure of the title compound with labeling and displacement ellipsoids drawn at the 50% probability level.

The asymmetric unit of the title compound, Co(NCS)_2_(C_3_H_6_N_2_S)_2_ (C_3_H_6_N_2_S = ethyl­ene­thio­urea), which crystallizes in space group *P*2_1_/*c*, consists of one crystallographically independent cobalt cation, two independent thio­cyanat anions and two independent ethyl­ene­thio­urea ligands with all atoms lying on general positions. The metal cations are fourfold coordinated (Table 1 ▸) by two N-bonding thio­cyanate anions and two ethyl­ene­thio­urea ligands in a slightly distorted tetra­hedral environment (Fig. 2 ▸). The C11 ethyl­ene­thio­urea ring is an envelope with atom C12 as the flap whereas the C21 ring is twisted about the C22—C23 bond. Therefore, this structure is completely different from that of the known isomer of Co(NCS)_2_(ethyl­ene­thio­urea) already reported in the literature, which consists of octa­hedrally cobalt cations that are linked into chains *via* pairs of μ-1,3-bridging thio­cyanato anions (Fig. 1 ▸).

Even if cobalt thio­cyanate compounds with an octa­hedral coordination and bridging anionic ligands represent the majority of structures, compounds with a tetra­hedral coordin­ation are also reported. These include, for example, Co(NCS)_2_(4-*N*,*N*′-di­methyl­amino­pyridine)_2_, which crystallizes in different polymorphic modifications [Neumann *et al.*, 2018 ▸ (refcode GIQPEE); Krebs *et al.*, 2021*b* ▸ (GIQPEE01 and GIQPEE02)] as well as Co(NCS)_2_(3-amino­pyridine (ANU­MII; Mautner *et al.*, 2021 ▸), Co(NCS)_2_(4-amino­pyridine)_2_ (UGUWEA; Sugiyama *et al.*, 2015 ▸) Co(NCS)_2_(4-vinyl­pyridine)_2_ (BOZJUW; Foxman & Mazurek, 1982 ▸) and Co(NCS)_2_(3-methyl­pyridine)_2_ (EYARIG; Boeckmann *et al.*, 2011 ▸).

## 3. Supra­molecular features

**Figure 3:**
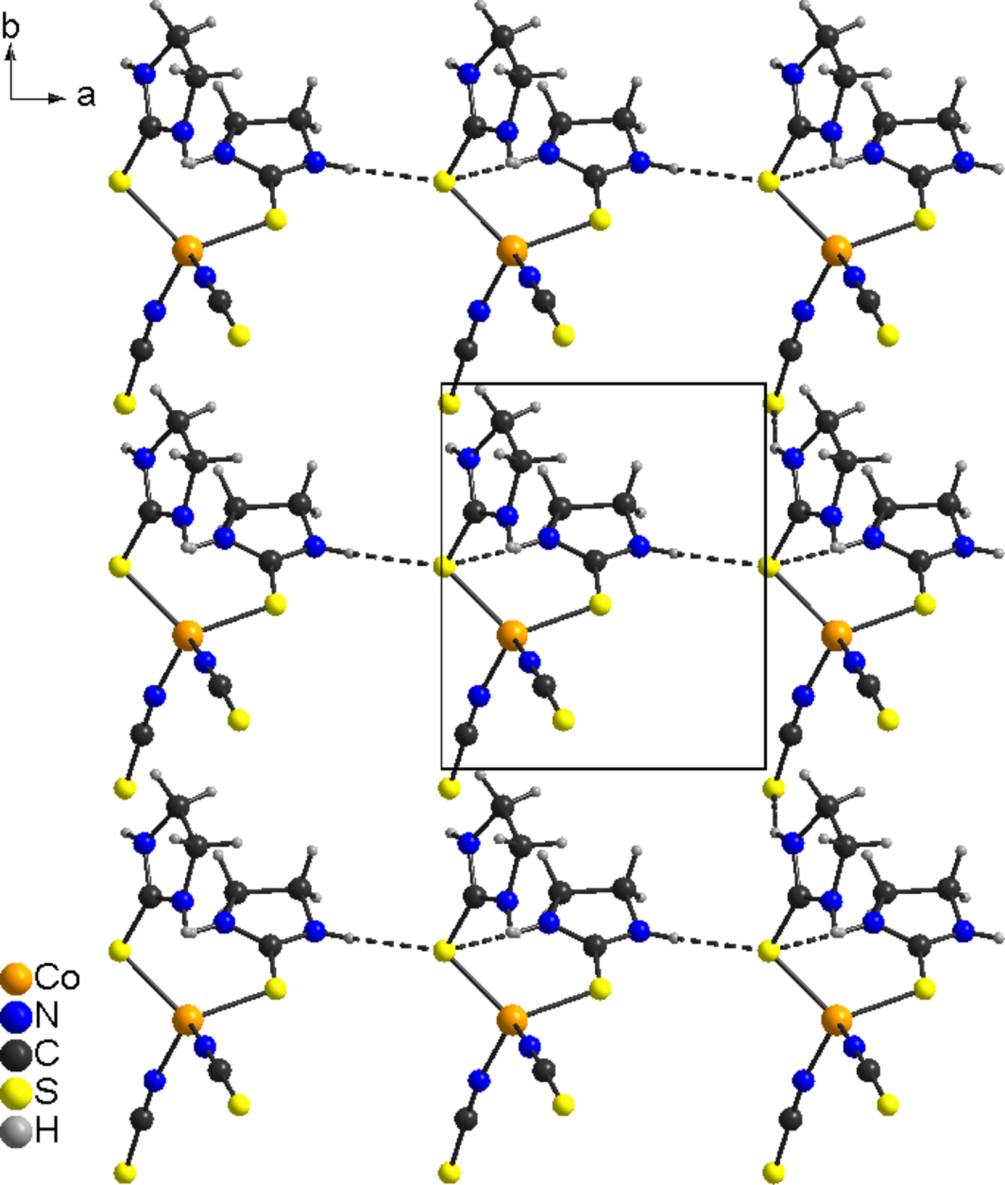
View onto a layer in the crystal structure of the title compound. Inter­molecular N—H⋯S hydrogen bonding is shown as dashed lines.

**Figure 4:**
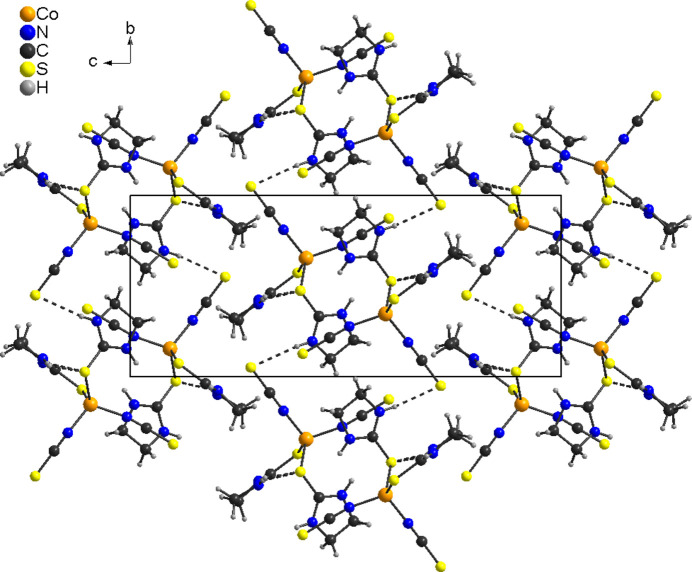
Crystal structure of the title compound in a view along the crystallographic *a*-axis direction. Inter­molecular N—H⋯S hydrogen bonding is shown as dashed lines.

In the extended structure of the title compound, a number of N—H⋯S inter­actions are observed (Table 2 ▸), with some of them at relatively short H⋯S distances and N—H⋯S angles close to linearity, indicating hydrogen bonding rather than incidental contacts (Table 2 ▸). This leads to the formation of chains, which propagate in the crystallographic *a*-axis direction (Fig. 3 ▸). These chains are further connected into layers that lie parallel to the *bc* plane (Fig. 4 ▸). Additional N—H⋯S inter­actions are found between the layers, but at long distances and angles far from 180°.

## 4. Physical characterization

**Figure 5:**
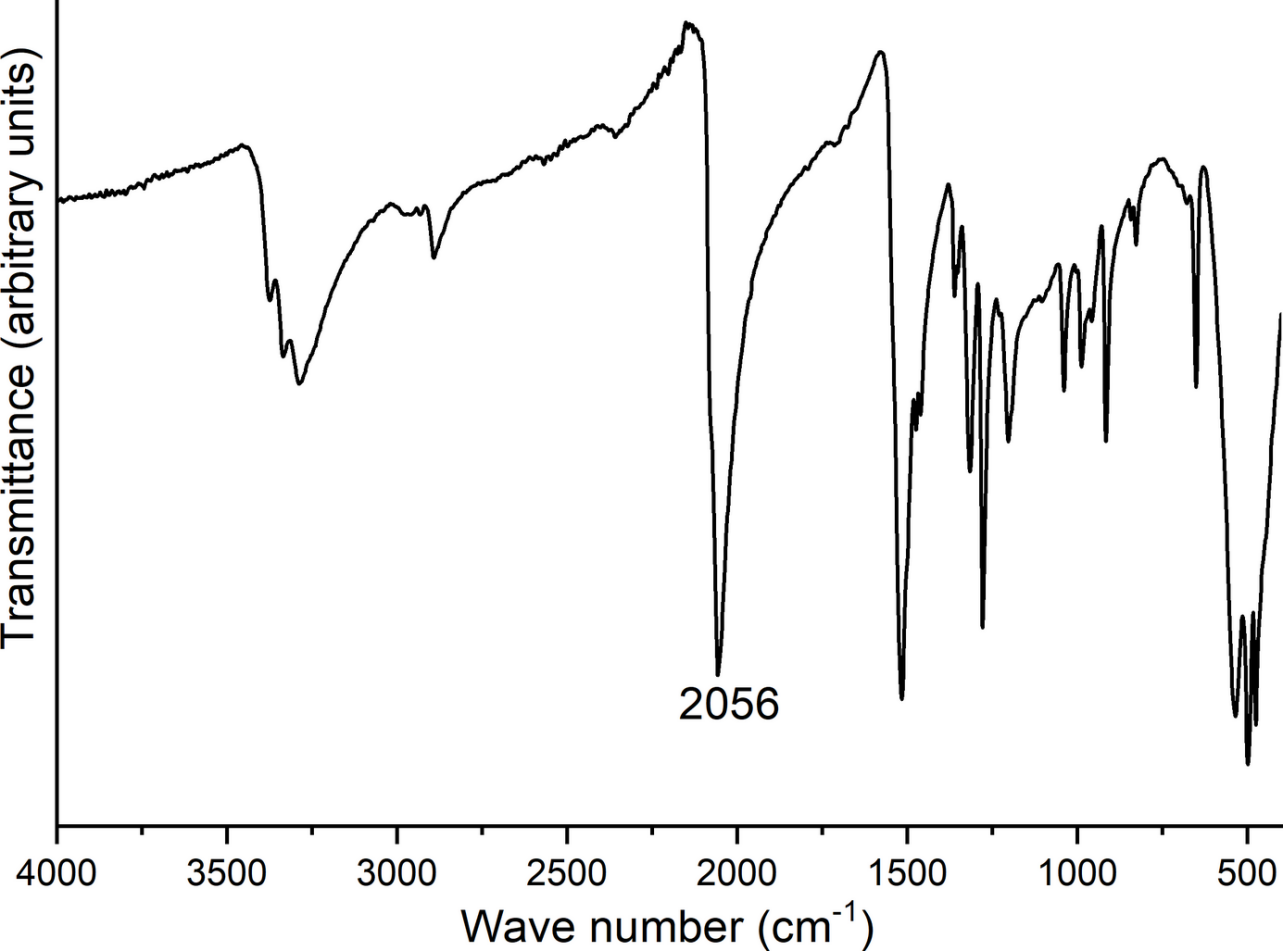
IR spectrum of the title compound. The value of the CN stretching vibration of the thio­cyanate anions is given.

The IR spectrum reveals that the CN stretching vibration of the thio­cyanate anions occurs at 2056 cm^−1^, in agreement with the presence of tetra­hedral coordination (Fig. 5 ▸). The bands between 3000 and 3500 cm^−1^ can be assigned to the N—H stretching vibration with this group involved in inter­molecular hydrogen bonding.

**Figure 6:**
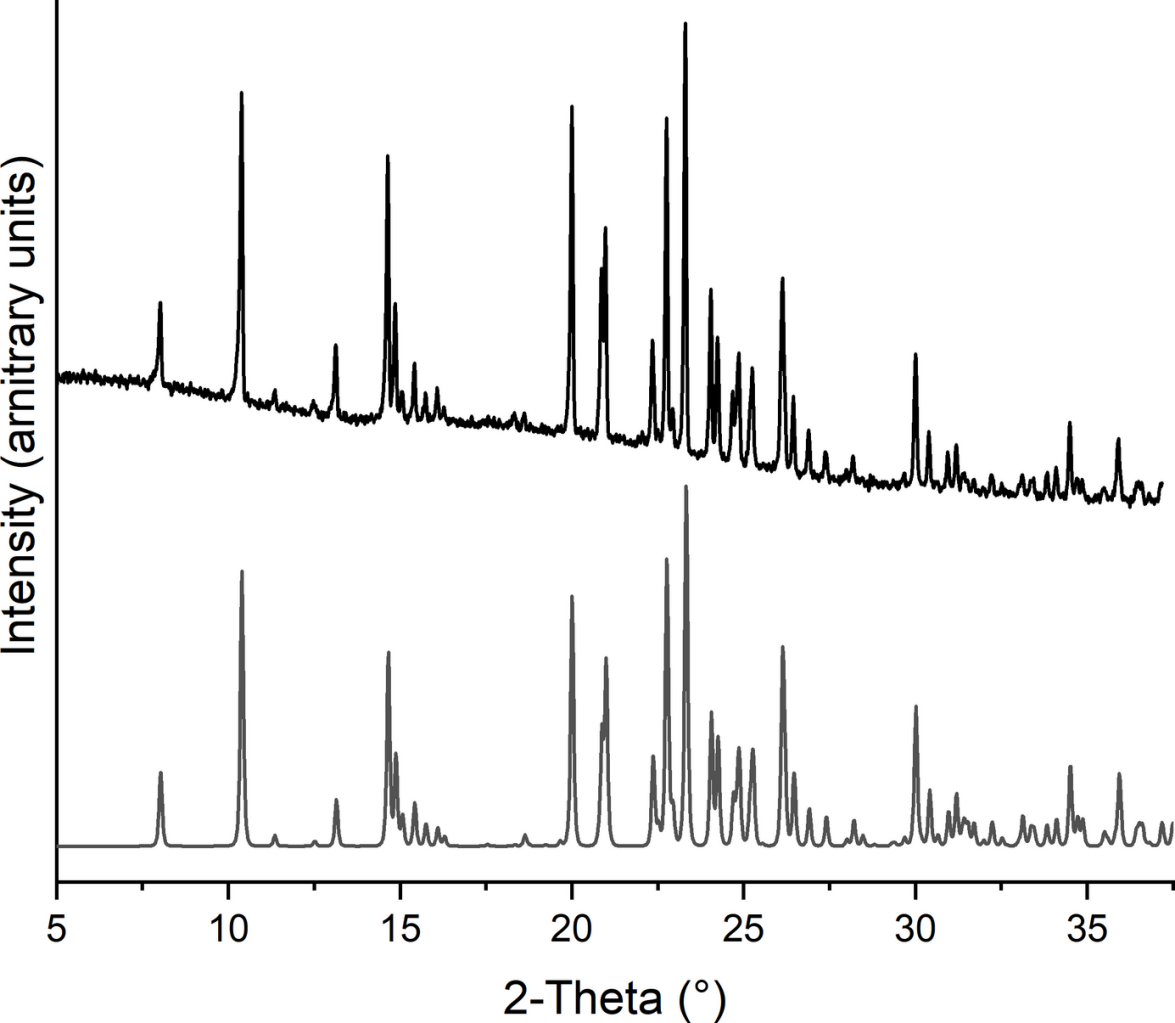
Experimental (top) and calculated (bottom)X-ray powder patterns of the title compound.

Comparison of the experiment X-ray powder pattern with that calculated using single crystal data reveal that a pure sample has been obtained (Fig. 6 ▸).

**Figure 7:**
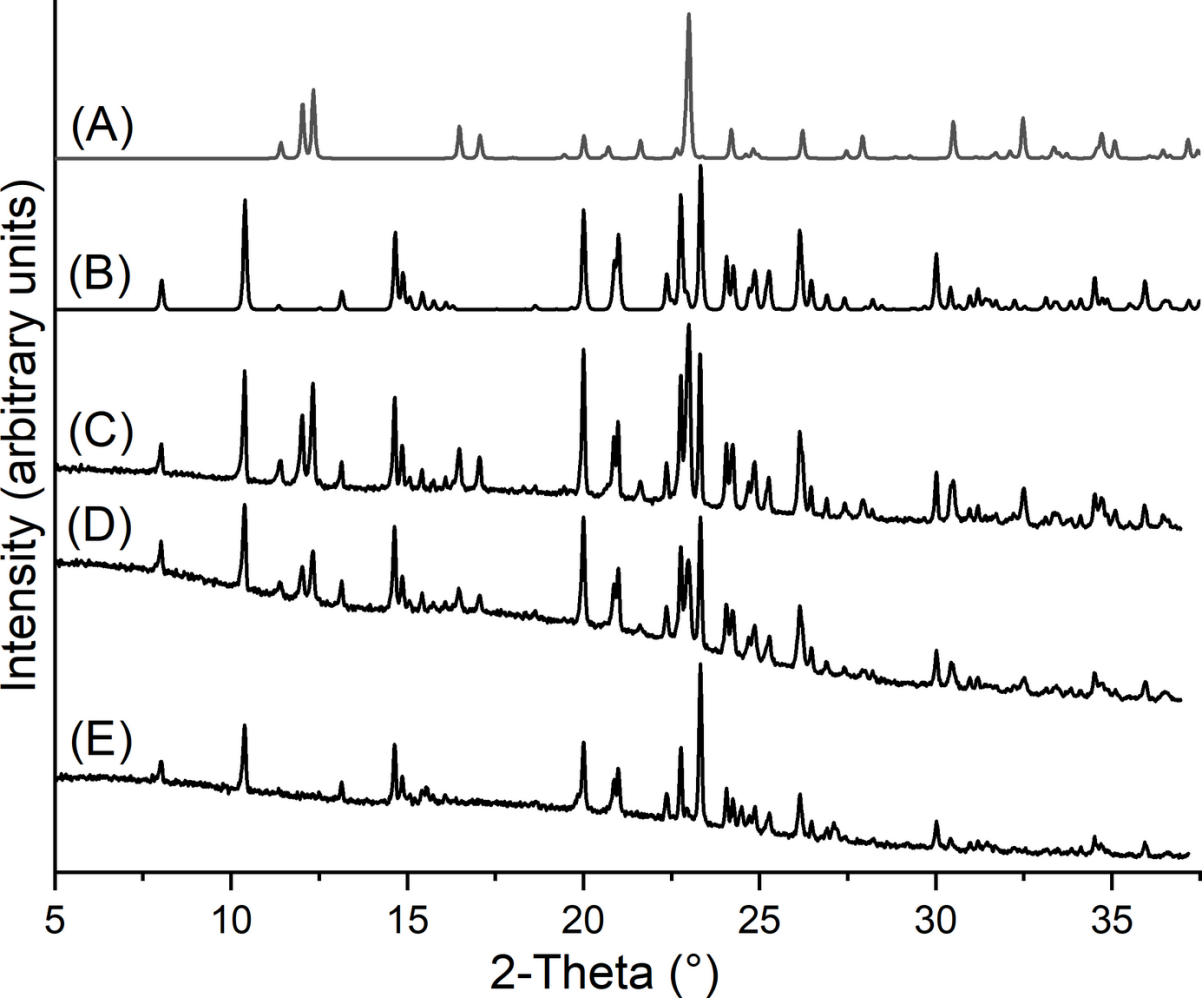
Calculated X-ray powder pattern of the chain isomer (A) and the title compound (B) as well as experimental pattern of a mixture of both isomers (C) and after stirring this mixture in ethanol for 1 d (D) and 4 d (E).

To investigate which of the two modifications of Co(NCS)_2_(C_3_H_6_N_2_S)_2_ is the thermodynamically stable form at room temperature, solvent-mediated conversion experiments were performed. In this experiment, a mixture of the two isomers with excess of solid was stirred in ethanol at room temperature and these residues were investigated by X-ray powder diffraction after one and four days (Fig. 7 ▸). This shows that the reflections of the chain isomer disappear completely, which proves that the title compound represents the thermodynamically stable isomer at room temperature, where the chain isomer is metastable.

## 5. Database survey

First of all it may be mentioned that beside the chain isomer of Co(NCS)_2_(C_3_H_6_N_2_S)_2_ (CSD refcode ZZZFAI01; Jochim *et al.*, 2020*a* ▸), another compound with same composition is reported in the Cambridge Structural Database [CSD Version 5.43, update of May 2026 (Groom *et al.*, 2016 ▸), search with CONQUEST (Bruno *et al.*, 2002 ▸)] for which only unit-cell parameters are reported (ZZZFAI; Nardelli & Chierici, 1958 ▸). According to the entry in the CSD, this compound should consist of discrete complexes but the unit-cell parameters are very similar to the chain isomer of this compound. Moreover, it is stated that this form crystallizes in the triclinic system with *Z* = 1, in which space group *P*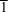 is impossible. The same authors also reported unit-cell parameters for the corresponding Mn and Zn compounds. The unit-cell parameters for the Mn compound (ZZZEZA; Nardelli & Chierici, 1958 ▸) are also very similar to those of the chain isomer, whereas the Zn compound is isotypic to the title complex (ZZZDUE; Nardelli & Chierici, 1958 ▸). Later, Nardelli and co-workers reported the crystal structure of Ni(NCS)_2_(C_3_H_6_N_2_S)_2_ (ESUNSC10; Nardelli *et al.*, 1966 ▸), which consists of chains and is isotypic to the chain isomer of the corresponding Co compound. The crystal structure of the Cd compound is also published and consists of chains, but is not isotypic to the Co and Ni compounds (ETCDTH; Calvaca *et al.*, 1960 ▸).

Finally, there are some compounds with Co(NCS)_2_ and other thio­urea derivatives as ligand reported in the CSD, including Co(NCS)_2_(tetra­methyl­thio­urea)_2_ (WUQTIO; Jochim *et al.*, 2020*b* ▸), Co(NCS)_2_(*N*,*N*′-di­methyl­thio­urea)_2_ (QUSZAI; Jochim *et al.*, 2020*c* ▸) and Co(NCS)_2_(1,3-di­cyclo­hexyl­thio­urea)_2_ (LAMPUO; Krebs *et al.*, 2022 ▸). All of these compounds consist of discrete complexes with a tetra­hedral coordination. However, in Co(NCS)_2_(thio­urea)_2_, the cobalt cations are linked by pairs of μ-1,3-bridging thio­cyanate into chains (LEHQAS; Rajarajan *et al.*, 2012 ▸).

## 6. Synthesis and crystallization

Cobalt thio­cyanate and ethyl­ene­thio­urea were purchased from Sigma-Aldrich. 87.6 mg (0.50 mmol) of Co(NCS)_2_ and 102.4 mg (1.0 mmol) of ethyl­ene­thio­urea were stirred in 3 ml of ethanol for 3 d. Then, 3 ml of *n*-heptane were added and stirred for another 10 min. The precipitate was filtered off, leading to a microcrystalline powder of the title compound. As the solvent slowly evaporated from the filtrate, blue block-shaped crystals suitable for single crystal X-ray diffraction were obtained.

The chain isomer of Co(NCS)_2_(C_3_H_6_N_2_S)_2_ used for the solvent-mediated conversion experiments was prepared according to literature procedures (Jochim *et al.*, 2020*a* ▸).

The IR spectroscopic measurements were performed with an ATI Mattson Genesis Series FTIR Spectrometer, control software: *WINFIRST*, from ATI Mattson in ATR mode. The PXRD measurements were performed with Cu *K*α_1_ radiation (λ = 1.540598 Å) using a Stoe Transmission Powder Diffraction System (STADI P) equipped with a MYTHEN 1K detector and a Johansson-type Ge(111) monochromator.

## 7. Refinement

Crystal data, data collection and structure refinement details are summarized in Table 3 ▸.The C-bound hydrogen atoms were positioned with idealized geometry and were refined isotropically with *U*_iso_(H) = 1.2 *U*_eq_(C) using a riding model. The N-bound hydrogen atoms were located in difference maps, their bond lengths set to ideal values and finally they were refined isotropically with *U*_iso_(H) = 1.2 *U*_eq_(N) using a riding model.

## Supplementary Material

Crystal structure: contains datablock(s) I. DOI: 10.1107/S2056989026007383/hb8234sup1.cif

Structure factors: contains datablock(s) I. DOI: 10.1107/S2056989026007383/hb8234Isup2.hkl

CCDC reference: 2574161

Additional supporting information:  crystallographic information; 3D view; checkCIF report

## Figures and Tables

**Table 1:** Selected geometric parameters (Å, °)

Co1—N1	1.9446 (15)	Co1—S11	2.3111 (5)
Co1—N2	1.9544 (16)	Co1—S21	2.3120 (5)
			
N1—Co1—N2	110.32 (7)	S11—Co1—S21	113.076 (19)
N1—Co1—S11	114.80 (5)	C1—N1—Co1	167.80 (15)
N2—Co1—S11	102.29 (5)	C2—N2—Co1	171.01 (15)
N1—Co1—S21	100.61 (5)	C11—S11—Co1	102.33 (6)
N2—Co1—S21	116.30 (5)	C21—S21—Co1	110.89 (6)

**Table 2:** Hydrogen-bond geometry (Å, °)

*D*—H⋯*A*	*D*—H	H⋯*A*	*D*⋯*A*	*D*—H⋯*A*
N11—H11⋯S2^i^	0.88	2.57	3.3693 (16)	152
N12—H12⋯S11^ii^	0.88	2.95	3.5571 (17)	128
N12—H12⋯S21^iii^	0.88	2.97	3.4542 (15)	116
C12—H12*A*⋯S21^iii^	0.99	2.91	3.4371 (19)	114
C13—H13*B*⋯S1^iii^	0.99	2.95	3.816 (2)	147
N21—H21⋯S11^iv^	0.88	2.74	3.5823 (16)	161
N22—H22⋯S2^v^	0.88	2.83	3.3560 (16)	120
N22—H22⋯S11	0.88	2.73	3.5346 (15)	152
C22—H22*A*⋯S2^i^	0.99	2.96	3.800 (2)	143

Symmetry codes: (i) 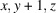; (ii) 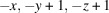; (iii) 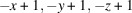; (iv) 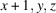; (v) 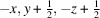.

**Table 3:** Experimental details

Crystal data	
Chemical formula	[Co(NCS)_2_(C_3_H_6_N_2_S)_2_]
*M*_r_	379.41
Crystal system, space group	Monoclinic, *P*2_1_/*c*
Temperature (K)	170
*a*, *b*, *c* (Å)	7.7636 (2), 9.1388 (3), 21.9230 (5)
β (°)	96.552 (2)
*V* (Å^3^)	1545.28 (7)
*Z*	4
Radiation type	Mo *K*α
μ (mm^−1^)	1.65
Crystal size (mm)	0.18 × 0.11 × 0.08

Data collection	
Diffractometer	Stoe IPDS2
Absorption correction	Numerical (*X-SHAPE* and *X-RED* 32; Stoe, 2008 ▸)
*T*_min_, *T*_max_	0.638, 0.798
No. of measured, independent and observed [*I* > 2σ(*I*)] reflections	21701, 3351, 3080
*R*_int_	0.022
(sin θ/λ)_max_ (Å^−1^)	0.639

Refinement	
*R*[*F*^2^ > 2σ(*F*^2^)], *wR*(*F*^2^), *S*	0.025, 0.062, 1.08
No. of reflections	3351
No. of parameters	172
H-atom treatment	H-atom parameters constrained
Δρ_max_, Δρ_min_ (e Å^−3^)	0.30, −0.23

Computer programs: *X-AREA* and *X-RED* (Stoe, 2008 ▸), *SHELXT* (Sheldrick, 2015*a* ▸), *SHELXL* (Sheldrick, 2015*b* ▸), *DIAMOND* (Brandenburg, 1999 ▸), *XP* in *SHELXTL-PC* (Sheldrick, 2008 ▸) and *publCIF* (Westrip, 2010 ▸).

## supplementary crystallographic information

### Bis(imidazolidine-2-thione-κS)dithiocyanatocobalt(II) Crystal data

**Table:**

[Co(NCS)_2_(C_3_H_6_N_2_S)_2_]	*F*(000) = 772
*M**_r_* = 379.41	*D*_x_ = 1.631 Mg m^−^^3^
Monoclinic, *P*2_1_/*c*	Mo *K*α radiation, λ = 0.71073 Å
*a* = 7.7636 (2) Å	Cell parameters from 8000 reflections
*b* = 9.1388 (3) Å	θ = 10.0–25.0°
*c* = 21.9230 (5) Å	µ = 1.65 mm^−^^1^
β = 96.552 (2)°	*T* = 170 K
*V* = 1545.28 (7) Å^3^	Block, blue
*Z* = 4	0.18 × 0.11 × 0.08 mm

### Bis(imidazolidine-2-thione-κS)dithiocyanatocobalt(II) Data collection

**Table:**

Stoe IPDS-2 diffractometer	3080 reflections with *I* > 2σ(*I*)
ω scans	*R*_int_ = 0.022
Absorption correction: numerical (X-Shape and X-Red 32; Stoe, 2008)	θ_max_ = 27.0°, θ_min_ = 1.9°
*T*_min_ = 0.638, *T*_max_ = 0.798	*h* = −9→9
21701 measured reflections	*k* = −11→11
3351 independent reflections	*l* = −28→28

### Bis(imidazolidine-2-thione-κS)dithiocyanatocobalt(II) Refinement

**Table:**

Refinement on *F*^2^	Primary atom site location: dual
Least-squares matrix: full	Hydrogen site location: mixed
*R*[*F*^2^ > 2σ(*F*^2^)] = 0.025	H-atom parameters constrained
*wR*(*F*^2^) = 0.062	*w* = 1/[σ^2^(*F*_o_^2^) + (0.031*P*)^2^ + 0.5882*P*] where *P* = (*F*_o_^2^ + 2*F*_c_^2^)/3
*S* = 1.08	(Δ/σ)_max_ = 0.001
3351 reflections	Δρ_max_ = 0.30 e Å^−^^3^
172 parameters	Δρ_min_ = −0.23 e Å^−^^3^
0 restraints	

### Bis(imidazolidine-2-thione-κS)dithiocyanatocobalt(II) Special details

**Table:**

Geometry. All esds (except the esd in the dihedral angle between two l.s. planes) are estimated using the full covariance matrix. The cell esds are taken into account individually in the estimation of esds in distances, angles and torsion angles; correlations between esds in cell parameters are only used when they are defined by crystal symmetry. An approximate (isotropic) treatment of cell esds is used for estimating esds involving l.s. planes.

### Bis(imidazolidine-2-thione-κS)dithiocyanatocobalt(II) Fractional atomic coordinates and isotropic or equivalent isotropic displacement parameters (Å^2^)

**Table:**

	*x*	*y*	*z*	*U*_iso_*/*U*_eq_	
Co1	0.21856 (3)	0.34622 (2)	0.40792 (2)	0.03252 (8)	
N1	0.2712 (2)	0.27718 (18)	0.49188 (7)	0.0412 (3)	
C1	0.3166 (2)	0.2152 (2)	0.53726 (8)	0.0372 (4)	
S1	0.37618 (8)	0.12604 (6)	0.59973 (2)	0.05231 (14)	
N2	0.1156 (2)	0.18903 (18)	0.35535 (7)	0.0413 (3)	
C2	0.0766 (2)	0.08932 (19)	0.32363 (7)	0.0336 (3)	
S2	0.02460 (6)	−0.05075 (5)	0.27987 (2)	0.03993 (11)	
S11	0.00832 (5)	0.52604 (5)	0.39459 (2)	0.03687 (11)	
C11	0.1030 (2)	0.66747 (18)	0.43786 (7)	0.0321 (3)	
N11	0.0839 (2)	0.80539 (18)	0.42222 (7)	0.0437 (4)	
H11	0.029316	0.832996	0.386690	0.052*	
N12	0.2017 (2)	0.65511 (16)	0.49075 (7)	0.0410 (3)	
H12	0.213882	0.575491	0.513402	0.049*	
C12	0.2443 (3)	0.7985 (2)	0.51792 (9)	0.0440 (4)	
H12A	0.369897	0.807233	0.531493	0.053*	
H12B	0.178507	0.817590	0.553222	0.053*	
C13	0.1886 (3)	0.9017 (2)	0.46461 (9)	0.0436 (4)	
H13A	0.119347	0.984540	0.477760	0.052*	
H13B	0.289889	0.940337	0.446111	0.052*	
S21	0.48883 (6)	0.42711 (5)	0.38813 (2)	0.03941 (11)	
C21	0.4724 (2)	0.53905 (18)	0.32552 (7)	0.0311 (3)	
N21	0.60888 (19)	0.57669 (18)	0.29779 (7)	0.0393 (3)	
H21	0.717127	0.559478	0.312620	0.047*	
N22	0.33055 (18)	0.59910 (18)	0.29709 (7)	0.0375 (3)	
H22	0.229503	0.573146	0.308204	0.045*	
C22	0.3662 (2)	0.6770 (2)	0.24139 (9)	0.0457 (4)	
H22A	0.311627	0.775101	0.238903	0.055*	
H22B	0.325286	0.620757	0.203928	0.055*	
C23	0.5628 (2)	0.6878 (3)	0.25054 (9)	0.0476 (5)	
H23A	0.613416	0.664837	0.212208	0.057*	
H23B	0.601404	0.786424	0.264968	0.057*	

### Bis(imidazolidine-2-thione-κS)dithiocyanatocobalt(II) Atomic displacement parameters (Å^2^)

**Table:**

	*U*^11^	*U*^22^	*U*^33^	*U*^12^	*U*^13^	*U*^23^
Co1	0.03490 (13)	0.03183 (12)	0.03132 (12)	−0.00233 (9)	0.00588 (9)	0.00202 (8)
N1	0.0486 (9)	0.0421 (8)	0.0333 (7)	−0.0018 (7)	0.0065 (6)	0.0033 (6)
C1	0.0409 (9)	0.0361 (9)	0.0356 (9)	−0.0001 (7)	0.0084 (7)	−0.0041 (7)
S1	0.0693 (3)	0.0490 (3)	0.0373 (2)	0.0069 (2)	0.0002 (2)	0.0068 (2)
N2	0.0449 (9)	0.0403 (8)	0.0390 (8)	−0.0043 (7)	0.0061 (6)	0.0006 (7)
C2	0.0322 (8)	0.0377 (9)	0.0313 (8)	0.0002 (7)	0.0048 (6)	0.0058 (7)
S2	0.0456 (2)	0.0387 (2)	0.0345 (2)	0.00118 (18)	0.00043 (17)	−0.00220 (17)
S11	0.0325 (2)	0.0404 (2)	0.0370 (2)	0.00086 (17)	0.00088 (16)	0.00048 (17)
C11	0.0289 (8)	0.0362 (8)	0.0318 (8)	0.0033 (6)	0.0070 (6)	0.0063 (6)
N11	0.0472 (9)	0.0384 (8)	0.0429 (8)	−0.0003 (7)	−0.0057 (7)	0.0120 (7)
N12	0.0519 (9)	0.0335 (8)	0.0355 (8)	0.0029 (7)	−0.0048 (6)	0.0055 (6)
C12	0.0503 (11)	0.0363 (9)	0.0436 (10)	0.0002 (8)	−0.0031 (8)	−0.0005 (8)
C13	0.0460 (10)	0.0342 (9)	0.0498 (10)	0.0016 (8)	0.0021 (8)	0.0028 (8)
S21	0.0319 (2)	0.0459 (2)	0.0396 (2)	−0.00271 (17)	0.00027 (16)	0.01317 (18)
C21	0.0307 (8)	0.0317 (8)	0.0309 (8)	−0.0011 (6)	0.0034 (6)	−0.0025 (6)
N21	0.0280 (7)	0.0483 (9)	0.0426 (8)	0.0028 (6)	0.0085 (6)	0.0102 (7)
N22	0.0280 (7)	0.0475 (8)	0.0376 (7)	0.0007 (6)	0.0065 (6)	0.0111 (6)
C22	0.0388 (10)	0.0556 (12)	0.0437 (10)	0.0054 (8)	0.0096 (8)	0.0184 (9)
C23	0.0401 (10)	0.0595 (12)	0.0447 (10)	0.0002 (9)	0.0110 (8)	0.0180 (9)

### Bis(imidazolidine-2-thione-κS)dithiocyanatocobalt(II) Geometric parameters (Å, º)

**Table:**

Co1—N1	1.9446 (15)	C12—H12A	0.9900
Co1—N2	1.9544 (16)	C12—H12B	0.9900
Co1—S11	2.3111 (5)	C13—H13A	0.9900
Co1—S21	2.3120 (5)	C13—H13B	0.9900
N1—C1	1.164 (2)	S21—C21	1.7050 (17)
C1—S1	1.6153 (18)	C21—N22	1.321 (2)
N2—C2	1.165 (2)	C21—N21	1.326 (2)
C2—S2	1.6228 (18)	N21—C23	1.466 (2)
S11—C11	1.7182 (18)	N21—H21	0.8800
C11—N11	1.310 (2)	N22—C22	1.467 (2)
C11—N12	1.320 (2)	N22—H22	0.8801
N11—C13	1.459 (2)	C22—C23	1.520 (3)
N11—H11	0.8800	C22—H22A	0.9900
N12—C12	1.462 (2)	C22—H22B	0.9900
N12—H12	0.8799	C23—H23A	0.9900
C12—C13	1.526 (3)	C23—H23B	0.9900
			
N1—Co1—N2	110.32 (7)	N11—C13—H13A	111.4
N1—Co1—S11	114.80 (5)	C12—C13—H13A	111.4
N2—Co1—S11	102.29 (5)	N11—C13—H13B	111.4
N1—Co1—S21	100.61 (5)	C12—C13—H13B	111.4
N2—Co1—S21	116.30 (5)	H13A—C13—H13B	109.3
S11—Co1—S21	113.076 (19)	C21—S21—Co1	110.89 (6)
C1—N1—Co1	167.80 (15)	N22—C21—N21	110.04 (15)
N1—C1—S1	178.59 (18)	N22—C21—S21	127.65 (13)
C2—N2—Co1	171.01 (15)	N21—C21—S21	122.30 (13)
N2—C2—S2	179.20 (17)	C21—N21—C23	111.12 (15)
C11—S11—Co1	102.33 (6)	C21—N21—H21	124.3
N11—C11—N12	110.46 (16)	C23—N21—H21	122.1
N11—C11—S11	123.40 (13)	C21—N22—C22	111.61 (14)
N12—C11—S11	126.14 (13)	C21—N22—H22	118.6
C11—N11—C13	112.06 (15)	C22—N22—H22	128.3
C11—N11—H11	122.4	N22—C22—C23	101.74 (14)
C13—N11—H11	124.6	N22—C22—H22A	111.4
C11—N12—C12	111.27 (15)	C23—C22—H22A	111.4
C11—N12—H12	125.5	N22—C22—H22B	111.4
C12—N12—H12	120.4	C23—C22—H22B	111.4
N12—C12—C13	102.19 (15)	H22A—C22—H22B	109.3
N12—C12—H12A	111.3	N21—C23—C22	102.05 (14)
C13—C12—H12A	111.3	N21—C23—H23A	111.4
N12—C12—H12B	111.3	C22—C23—H23A	111.4
C13—C12—H12B	111.3	N21—C23—H23B	111.4
H12A—C12—H12B	109.2	C22—C23—H23B	111.4
N11—C13—C12	101.73 (15)	H23A—C23—H23B	109.2
			
Co1—S11—C11—N11	143.67 (14)	Co1—S21—C21—N22	12.57 (18)
Co1—S11—C11—N12	−37.17 (16)	Co1—S21—C21—N21	−166.68 (13)
N12—C11—N11—C13	5.1 (2)	N22—C21—N21—C23	7.9 (2)
S11—C11—N11—C13	−175.63 (13)	S21—C21—N21—C23	−172.69 (14)
N11—C11—N12—C12	5.5 (2)	N21—C21—N22—C22	4.9 (2)
S11—C11—N12—C12	−173.74 (14)	S21—C21—N22—C22	−174.42 (14)
C11—N12—C12—C13	−12.9 (2)	C21—N22—C22—C23	−14.7 (2)
C11—N11—C13—C12	−12.6 (2)	C21—N21—C23—C22	−16.5 (2)
N12—C12—C13—N11	14.3 (2)	N22—C22—C23—N21	17.5 (2)

### Bis(imidazolidine-2-thione-κS)dithiocyanatocobalt(II) Hydrogen-bond geometry (Å, º)

**Table:**

*D*—H···*A*	*D*—H	H···*A*	*D*···*A*	*D*—H···*A*
N11—H11···S2^i^	0.88	2.57	3.3693 (16)	152
N12—H12···S11^ii^	0.88	2.95	3.5571 (17)	128
N12—H12···S21^iii^	0.88	2.97	3.4542 (15)	116
C12—H12*A*···S21^iii^	0.99	2.91	3.4371 (19)	114
C13—H13*B*···S1^iii^	0.99	2.95	3.816 (2)	147
N21—H21···S11^iv^	0.88	2.74	3.5823 (16)	161
N22—H22···S2^v^	0.88	2.83	3.3560 (16)	120
N22—H22···S11	0.88	2.73	3.5346 (15)	152
C22—H22*A*···S2^i^	0.99	2.96	3.800 (2)	143

Symmetry codes: (i) *x*, *y*+1, *z*; (ii) −*x*, −*y*+1, −*z*+1; (iii) −*x*+1, −*y*+1, −*z*+1; (iv) *x*+1, *y*, *z*; (v) −*x*, *y*+1/2, −*z*+1/2.

## References

[bb1] Barnett, S. A., Blake, A. J., Champness, N. R. & Wilson, C. (2002). *Chem. Commun.* pp. 1640–1641. 10.1039/b203661d 12170821

[bb2] Boeckmann, B., Reimer, B. & Näther, C. (2011). *Z. Naturforsch.* **66**, 819–827.

[bb3] Böhme, M., Jochim, A., Rams, M., Lohmiller, T., Suckert, S., Schnegg, A., Plass, W. & Näther, C. (2020). *Inorg. Chem.* **59**, 5325–5338. 10.1021/acs.inorgchem.9b03357 32091883

[bb4] Braga, D. & Grepioni, F. (2000). *Chem. Soc. Rev.* **29**, 229–238.

[bb5] Brandenburg, K. (1999). *DIAMOND*. Crystal Impact GbR, Bonn, Germany.

[bb6] Bruno, I. J., Cole, J. C., Edgington, P. R., Kessler, M., Macrae, C. F., McCabe, P., Pearson, J. & Taylor, R. (2002). *Acta Cryst.* B**58**, 389–397. 10.1107/s0108768102003324 12037360

[bb7] Cavalca, L., Nardelli, M. & Fava, G. (1960). *Acta Cryst.* **13**, 125–130.

[bb8] Foxman, B. M. & Mazurek, H. (1982). *Inorg. Chim. Acta* **59**, 231–235.

[bb9] Groom, C. R., Bruno, I. J., Lightfoot, M. P. & Ward, S. C. (2016). *Acta Cryst.* B**72**, 171–179. 10.1107/S2052520616003954 PMC4822653 27048719

[bb10] Jochim, A., Lohmiller, T., Rams, M., Böhme, M., Ceglarska, M., Schnegg, A., Plass, W. & Näther, C. (2020*a*). *Inorg. Chem.* **59**, 8971–8982. 10.1021/acs.inorgchem.0c00815 32551545

[bb11] Jochim, A., Radulovic, R., Jess, I. & Näther, C. (2020*b*). *Acta Cryst.* E**76**, 1373–1377. 10.1107/S205698902001021X PMC7405571 32844033

[bb12] Jochim, A., Radulovic, R., Jess, I. & Näther, C. (2020*c*). *Acta Cryst.* E**76**, 1476–1481. 10.1107/S2056989020011111 PMC7472772 32939303

[bb13] Krebs, C., Ceglarska, M. & Näther, C. (2021*a*). *Z. Anorg. Allge Chem.* **647**, 552–559.

[bb14] Krebs, C., Jess, I. & Näther, C. (2021*b*). *Acta Cryst.* E**77**, 1120–1125. 10.1107/S2056989021010422 PMC8587972 34868648

[bb15] Krebs, C., Jess, I. & Näther, C. (2022). *Acta Cryst.* E**78**, 71–75. 10.1107/S205698902101327X PMC8739206 35079428

[bb16] Mautner, F. A., Jantscher, P. V., Fischer, R. C., Torvisco, A., Reichmann, K. & Massoud, S. S. (2021). *Transition Met. Chem.* **46**, 191–200.

[bb17] Mautner, F. A., Traber, M., Fischer, R. C., Torvisco, A., Reichmann, K., Speed, S., Vicente, R. & Massoud, S. S. (2018). *Polyhedron* **154**, 436–442.

[bb18] Moulton, B. & Zaworotko, M. J. (2001). *Chem. Rev.* **101**, 1629–1658. 10.1021/cr9900432 11709994

[bb19] Nardelli, M. & Chierici, I. (1958). *Gazz. Chim. Ital.* **88**, 248–258.

[bb20] Nardelli, M., Gasparri, G. F., Musatti, A. & Manfredotti, A. (1966). *Acta Cryst.* **21**, 910–919.

[bb21] Neumann, T., Jess, I., Pielnhofer, F. & Näther, C. (2018). *Eur. J. Inorg. Chem.* pp. 4972–4981.

[bb22] Neumann, T., Ceglarska, M., Germann, L. S., Rams, M., Dinnebier, R. E., Suckert, S., Jess, I. & Näther, C. (2018). *Inorg. Chem.* **57**, 3305–3314. 10.1021/acs.inorgchem.8b00092 29505252

[bb40] Rajarajan, K., Sendil Kumar, K., Ramesh, V., Shihabuddeen, V. & Murugavel, S. (2012). *Acta Cryst.* E**68**, m1125–m1126. 10.1107/S1600536812033193 PMC3414167 22904774

[bb23] Rams, M., Jochim, A., Böhme, M., Lohmiller, T., Ceglarska, M., Rams, M. M., Schnegg, A., Plass, W. & Näther, C. (2020). *Chem. Eur. J.* **26**, 2837–2851. 10.1002/chem.201903924 PMC7078958 31702081

[bb24] Sheldrick, G. M. (2008). *Acta Cryst.* A**64**, 112–122. 10.1107/S0108767307043930 18156677

[bb25] Sheldrick, G. M. (2015*a*). *Acta Cryst.* A**71**, 3–8.

[bb26] Sheldrick, G. M. (2015*b*). *Acta Cryst.* C**71**, 3–8.

[bb27] Stoe (2008). *X-AREA*, *X-RED32* and *X-SHAPE*. Stoe & Cie, Darmstadt, Germany.

[bb28] Suckert, S., Rams, M., Böhme, M., Germann, L. S., Dinnebier, R. E., Plass, W., Werner, J. & Näther, C. (2016). *Dalton Trans.* **45**, 18190–18201. 10.1039/c6dt03752f 27796392

[bb29] Sugiyama, A., Sekine, A. & Uekusa, H. (2015). *X-ray Anal. Online.* **31**, 27–28.

[bb30] Wellm, C., Neumann, T., Gallo, G., Dziubyna, A. M., Rams, M., Dinnebier, R. E. & Näther, C. (2020). *Cryst. Growth Des.* **20**, 3374–3385.

[bb31] Westrip, S. P. (2010). *J. Appl. Cryst.* **43**, 920–925.

[bb32] Wöhlert, S., Fic, T., Tomkowicz, Z., Ebbinghaus, S. G., Rams, M., Haase, W. & Näther, C. (2013). *Inorg. Chem.* **52**, 12947–12957. 10.1021/ic4012235 24171470

[bb33] Wöhlert, S., Ruschewitz, U. & Näther, C. (2012). *Cryst. Growth Des.* **12**, 2715–2718.

